# A Novel and Conserved *Plasmodium* Sporozoite Membrane Protein SPELD is Required for Maturation of Exo-erythrocytic Forms

**DOI:** 10.1038/srep40407

**Published:** 2017-01-09

**Authors:** Faisal Mohammed Abdul Al-Nihmi, Surendra Kumar Kolli, Segireddy Rameswara Reddy, Babu S. Mastan, Jyothi Togiri, Mulaka Maruthi, Roshni Gupta, Puran Singh Sijwali, Satish Mishra, Kota Arun Kumar

**Affiliations:** 1 Department of Animal Biology, School of Life Sciences, University of Hyderabad, Hyderabad 500046, India; 2 Division of Parasitology, CSIR-Central Drug Research Institute, Lucknow 226031, India; 3 CSIR-Centre for Cellular and Molecular Biology, Habsiguda, Uppal Road, Hyderabad 500007, India

## Abstract

*Plasmodium* sporozoites are the infective forms of malaria parasite to vertebrate host and undergo dramatic changes in their transcriptional repertoire during maturation in mosquito salivary glands. We report here the role of a novel and conserved *Plasmodium berghei* protein encoded by *PBANKA_091090* in maturation of Exo-erythrocytic Forms (EEFs) and designate it as Sporozoite surface Protein Essential for Liver stage Development (PbSPELD). *PBANKA_091090* was previously annotated as *PB402615.00.0* and its transcript was recovered at maximal frequency in the Serial Analysis of the Gene Expression (SAGE) of *Plasmodium berghei* salivary gland sporozoites. An orthologue of this transcript was independently identified in *Plasmodium vivax* sporozoite microarrays and was designated as Sporozoite Conserved Orthologous Transcript-2 (*scot-2*). Functional characterization through reverse genetics revealed that PbSPELD is essential for *Plasmodium* liver stage maturation. mCherry transgenic of PbSPELD localized the protein to plasma membrane of sporozoites and early EEFs. Global microarray analysis of *pbspeld* ko revealed EEF attenuation being associated with down regulation of genes central to general transcription, cell cycle, proteosome and cadherin signaling. *pbspeld* mutant EEFs induced pre-erythrocytic immunity with 50% protective efficacy. Our studies have implications for attenuating the human *Plasmodium* liver stages by targeting SPELD locus.

Malaria is an infectious disease caused by a protozoan parasite that belongs to the genus *Plasmodium*. In 2013 alone, the reported mortality associated with malaria was about 854,586 cases^1^. Malaria is transmitted to humans by the bite of a female *Anopheles* mosquito that injects sporozoites into the skin of the host^2^. The sporozoites make their way to the liver where they transform into EEFs or liver stages. Following asexual exo-erythrocytic schizogony, the hepatic merozoites are released into the blood stream to initiate an erythrocytic cycle. During this phase, a proportion of parasites undergo differentiation to sexual forms called as gametocytes. When a female *Anopheles* mosquito ingests these gametocytes during the process of obtaining a blood meal, the male and female gametes fuse and result in the formation of a zygote. The zygote transforms into a motile ookinete that breaches the mosquito midgut epithelium and settles on hemocoel side of gut. The end product of sexual reproduction are the oocysts that undergo sporulation and upon rupture, release sporozoites into hemocoel^3^. The sporozoites migrate to the salivary glands and wait for transmission to humans when the mosquito probes for a blood meal.

High throughput methods of gene expression analysis have offered an insight in understanding the malaria parasite biology and allowed the appreciation of stage specifically regulated gene expression in modulating the infectivity or virulence of parasites^4^,^5^,^6^,^7^. Significant changes occur in the transcriptional repertoire of salivary gland sporozoites rendering them highly infective for hepatocytes^8^. The first comprehensive transcriptomic analysis of sporozoites^9^ opened the possibility of understanding the regulation of *Plasmodium* gene expression in mosquito stages that further led to investigating the differential gene expression between salivary gland sporozoite stages and other stages of *Plasmodium* using techniques like suppressive subtraction hybridization (SSH) to identify the transcripts uniquely upregulated in sporozoite stages. These studies led to the discovery of UIS genes^8^ and S genes^10^. Other independent studies have reported the analysis of expressed sequence tag (EST) data sets of salivary glands sporozoites and identified unique transcripts encoding secretory and membrane associated proteins important for sporozoites infectivity to hepatocytes^11^,^12^. Transcriptome of salivary gland sporozoite stage was also studied by Serial Analysis of Gene Expression (SAGE)^13^. In this analysis, 123 genes were identified out of which 66 were reported for the first time and were designated as SIS genes (new Sporozoite expressed genes Identified by SAGE)^13^. Clearly, the protein products encoded by these upregulated transcripts in sporozoite stage were important for regulation of parasite latency in salivary glands^14^, or functions central to motility^15^,^16^,^17^, cell traversal^11^,^12^,^18^,^19^, infectivity to hepatocytes^20^,^21^ liver stage development^22^,^23^,^24^,^25^,^26^,^27^ and egress^28^.

One of the highly recovered tags present in maximal abundance in the SAGE transcriptome analysis belonged to a gene that was newly annotated as *PB402615.00.0* in that study^13^. The sequence of *PB402615.00.0* aligned with the *P. yoelli* ESTs obtained from sporozoites and axenically developing EEF stages and its identified *P. yoelli* orthologue was *PY02432*. The current annotation of *PB402615.00.0* in PlasmoDB is *PBANKA_091090* and it encodes PbSPELD, as identified in this study. Based on the transcript abundance, *pbspeld* was grouped in a category^13^ that clustered frequency wise with previously described transcripts: *UIS4, UIS7*^8^, *S23*^10^ and *TRAP*^9^ discovered through independently generated subtraction or cDNA libraries generated from salivary gland sporozoite stages. However, no functional investigation of *pbspeld* has been performed till to date. Aiming to identify the role of its product in sporozoite commitment to hepatocytes and in EEF development, we generated *pbspeld* ko and demonstrate for the first time the role of this gene product in EEF maturation, a function that is consistent with the expression of PbSPELD in mosquito sporozoite stage and very early liver stage. We further demonstrate that attenuated *pbspeld* mutants manifested an altered global transcriptional profile at 36 h where 552 genes were down regulated and 1201 genes were upregulated. We report the ability of *pbspeld* ko EEFs in generating both humoral and cell mediated immunity that conferred significant protection against sporozoite challenge.

## Results

### *pbspeld* is a single copy gene unique to *Plasmodium* species and contains transmembrane domain and tyrosine rich motifs

**Figure 1:**
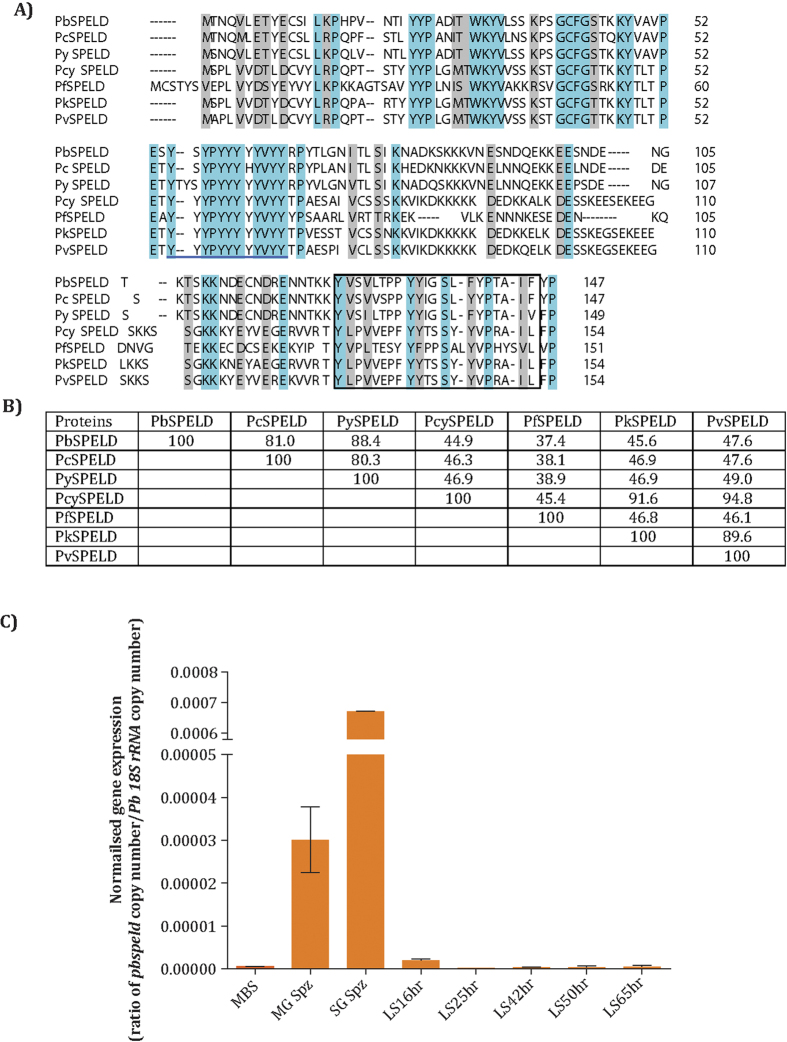
Alignment of amino acid sequence of SPELD orthologues and analysis of *pbspeld* gene expression across *Plasmodium* life cycle stages. (**A**) The amino acid sequence of PbSPELD was aligned with other *Plasmodium* orthologues *P. chabaudi*, PCHAS_0712200; *P. yoelii*, PY17X_0912300; *P. cynomolgi*, PCYB_094370; *P. falciparum*, PF3D7_1137800; *P. knowlesi*, PKNH_0936000 and *P. vivax* PVX_092505 using the Clustal Omega program. The conserved residues are shaded turquoise and physico-chemically similar residues are shaded grey. All orthologues contain a tyrosine-rich domain (underlined) and a putative transmembrane region (boxed). (**B**) The table shows amino acid identities between the indicated proteins. (**C**) The expression of *pbspeld* across *P. berghei* life cycle stages. The gene expression was analysed by quantitative real time PCR that revealed highest gene expression in the salivary gland sporozoites (SG Spz) followed by day 14 midgut sporozoites (MG Spz). The normalized data was expressed as a ratio of absolute copy numbers of *pbspeld* versus *Pb18srRNA* (internal control) for each stage of the *Plasmodium* life cycle. Ring: Ring stages, MBS: mixed blood stages, MG Spz: Midgut sporozoites, SG Spz: salivary gland sporozoites, LS16H: Liver stage 16 h, LS25H: Liver stage 25 h, LS42H: Liver stage 42 h, LS50H: Liver stage 50 h, LS65H: Liver stage 65 h.

The *PBANKA_091090* gene that codes for PbSPELD was identified as the most abundantly expressed sequence tag in the SAGE transcriptome analysis^13^. A BLAST search of the PlasmoDB using PbSPELD as query identified single homologs in other *Plasmodium* species (Fig. 1A), indicating that it is a single copy gene. Sequence analysis of homologues revealed that while *P. vivax, P. cynomolgi*, and *P. knowlesi* SPELD proteins share maximum identity; the rodent malaria parasite SPELD proteins are more closely related (81–88% sequence identity). Searches of Pfam (http://pfam.xfam.org/) and conserved domain database (http://www.ncbi.nlm.nih.gov/Structure/cdd/wrpsb.cgi) using PbSPELD did not identify any conserved domain; BLAST search of the NCBI database of “non-redundant protein sequences” also did not yield any homolog, indicating that SPELD proteins are unique to *Plasmodium*. PbSPELD sequence was analysed for the presence of transmembrane regions using multiple programs (TMHMM, TMpred, DAS, TopPred2), which predicted a single transmembrane region that is shared by all SPELD proteins (Fig. 1A). The SPELD proteins contain a tyrosine-rich domain, and the most abundant amino acid is tyrosine (*Pb*SPELD 13.6%, *Pc* SPELD 13.6%, *Py* SPELD 13.4%, *Pcyn* SPELD 14.9%, *Pf* SPELD 17.2%, *Pk* SPELD 14.9%, *Pv* SPELD 14.9%). Amino acid identities between the indicated proteins are shown in Fig. 1B. *In silico* analysis of SPELD protein sequence across rodent and human *Plasmodium* species is described in supplementary methods.

### *pbspeld* transcripts are highly expressed in salivary gland sporozoite stage

Quantification of *pbspeld* transcript by qRT PCR revealed maximal expression in salivary gland sporozoite stage followed by day 14 oocyst stage. The normalized gene expression for each stage as shown in Fig. 1C was obtained as a ratio of copy number of *pbspeld*/*Pb18SrRNA*. These studies were consistent with the maximal expression of *PBANKA_091090* in transcriptomic analysis of *P. berghei* infected salivary gland sporozoites performed by SAGE analysis^13^.

### Generation of *pbspeld* ko and *P. berghei* ANKA WT GFP transgenic line

**Figure 2:**
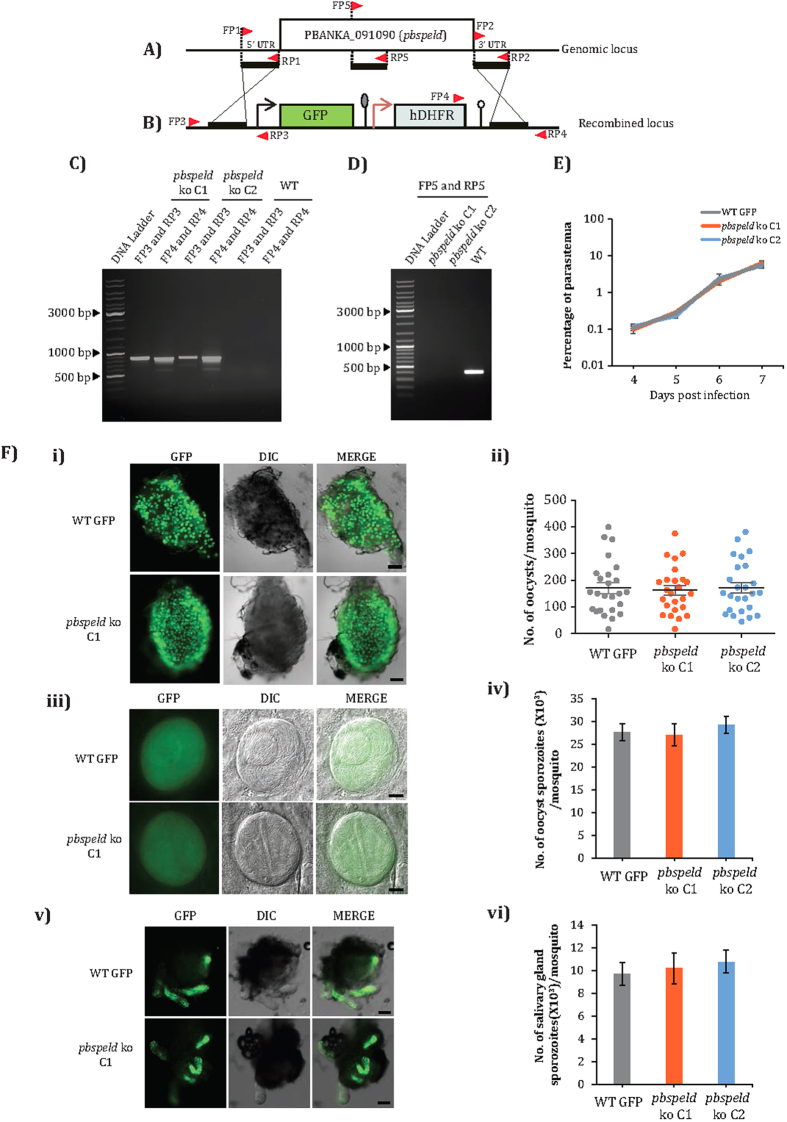
Generation of *pbspeld* ko parasite line and its phenotypic analysis in asexual blood stages and mosquito stages. (**A**) Genomic locus of *pbspeld* showing 5′ and 3′ regions and position of the primers are indicated by arrows. (**B**) Cloning of 5′ and 3′ regions in targeting vector pBC-GFP-hDHFR. (**C**) Agarose gel showing products of diagnostic PCR with primer set FP3/RP3 and FP4/RP4 indicating correct 5′ and 3′ integration respectively in *pbspeld* ko clone C1 and C2. No product was amplified from genomic DNA of WT parasites using same set of primers. (**D**) Agarose gel showing 395 bp product amplified with primer set FP5/RP5 within the *pbspeld* ORF from the genomic DNA of WT GFP parasite but not from *pbspeld ko* clone C1 and C2, confirming successful clonal dilution of ko line. (**E**) Asexual stages of *pbspeld* ko clone C1 and C2 propagate at similar rates as WT GFP parasites. (**F**) Mosquito stages of *pbspeld* ko does not show any defect in oocyst development, sporulation and in its ability to migrate to salivary glands. (i) Representative picture of mosquito midguts showing oocyst from WT GFP parasites and one of the *pbspeld* ko clone C1. Scale bar 200 μm. (ii) The frequency of oocyst per mosquito (n = 25/per group) in WT GFP, *pbspeld* ko clone C1 and C2. In each group, the horizontal line indicates mean ± SEM. (iii) Representative picture of oocyst from WT GFP and one of the *pbspeld* ko clone C1. Scale bar 20 μm. (iv) The average number of oocyst sporozoite from WT GFP, *pbspeld* ko clone C1 and C2. Values are mean ± SD (from 3 independent experiments, n = 25 mosquitoes/experiment). (v) Dissected salivary glands showing similar sporozoite loads from WT GFP and one of the *pbspeld* ko clone C1. Scale bar 200 μm. vi) The average number of salivary gland sporozoite from WT GFP, *pbspeld* ko clone C1 and C2. Values are mean ± SD (from 3 independent experiments, n = 25 mosquitoes/experiment).

To determine the function of *pbspeld* in the *Plasmodium* life cycle, its locus was successfully disrupted yielding a ko line. To achieve a double cross over (DCO) homologous recombination to replace *pbspeld* locus (Fig. 2A), the 5′ and 3′ flanking sequences were cloned on either ends of the *GFP-hDHFR* cassette and the targeting construct was electroporated into the schizont stage. The recombined locus has a *GFP-hDHFR* cassette in place of target gene (Fig. 2B). Genomic DNA was isolated from drug resistant GFP positive parasites and correct site specific integration was confirmed by primers designed at sites beyond recombination. Following limiting dilution, a diagnostic PCR was carried out from two clones- C1 and C2 obtained from two independent transfections that generated product of 863 bp amplified using primer set FP3/RP3 and product of 825 bp using primer set FP4/RP4 thus confirming correct 5′ and 3′ integrations (Fig. 2C). The same set of primers did not amplify product from WT genomic DNA (Fig. 2C). The clones C1 and C2 were further confirmed for the absence of *pbspeld* gene by performing a diagnostic PCR using primer set FP5/RP5 that amplified a product of 869 bp only from WT genomic DNA (Fig. 2D). For systematic comparison of the *pbspeld* ko phenotype at all life cycle stages with WT parasites, we generated a *Pb* ANKA WT GFP transgenic parasite line by integrating the *GFP-hDHFR* cassette at the non-essential *P230p* locus (Supplementary Fig. S1 and Supplementary method).

### *pbspeld* is not essential for asexual blood stage propagation and development of mosquito stages

To monitor if *pbspeld* depletion affected asexual blood stage development, two groups of BALB/c mice (3 mice/group) were intravenously injected with 10^3^ infected RBCs of either WT GFP or clones C1 and C2 and the asexual blood stage propagation was monitored for 7 days by making Giemsa stained blood smears, on a daily basis. Both C1 and C2 clones propagated at similar rates as WT GFP parasites and showed all stages of asexual forms and sexual gametocyte stages. These observations revealed a non-essential role of PbSPELD in asexual blood propagation or commitment to form the gametocytes (Fig. 2E).

Transmission of *pbspeld* ko to mosquitoes resulted in formation of oocysts (day 14) at numbers comparable to those formed by the WT parasites. Shown in Fig. 2F(i) is a representative mosquito midgut harbouring oocysts derived from WT and clone C1 and Fig. 2F(ii) shows the frequency of the oocysts in clone C1 and C2 as compared to WT GFP line. The sporulation pattern inside oocyst in clone C1 was comparable to oocyst of the WT GFP line (Fig. 2F iii). The nearly similar oocyst sporozoite numbers recorded from clones C1 and C2 as compared to WT GFP revealed normal development of sporozoites in both mutant lines (Fig. 2F iv). The sporozoite load in intact salivary gland harbouring clone C1 was comparable to WT GFP (Fig. 2F v). The sporozoite numbers recorded from disrupted salivary glands containing clones C1 and C2 were also comparable to WT GFP (Fig. 2F vi). We conclude from these results that PbSPELD does not have a role in the developmental stages of *Plasmodium* that occur with in the *Anopheles* mosquito.

### *pbspeld* ko sporozoites fail to initiate blood stage infection when malaria is transmitted by mosquito bite

Transmission of *pbspeld* ko sporozoites through mosquito bite did not initiate blood stage infection in four and two independent experiments performed respectively with clone C1 and C2 (Table 1). All blood meal positive mosquitoes that were used for transmission were dissected and majority of them had high loads of sporozoites in the salivary glands. Thus lack of break through infection was not due to absence of salivary gland sporozoites in the batch of mosquitoes used for transmission experiments. However high doses (2 × 10^4^) of *pbspeld* ko sporozoites delivered through intravenously route led to occasional break through infection in both clones with a pre patent period of 9 days versus of 3.5 days for WT GFP line (Table 2). Since the delay of 1 day patency correlates to a 10 fold reduction in liver stage burden, the difference in patencies of *pbspeld* ko and WT parasites implicates a 10^5^ fold less burden of mutants as compared to WT^29^.

### The delayed prepatency in *pbspeld* ko is not associated with their inability to invade hepatocytes but due to growth arrest at mid liver stage development

**Figure 3:**
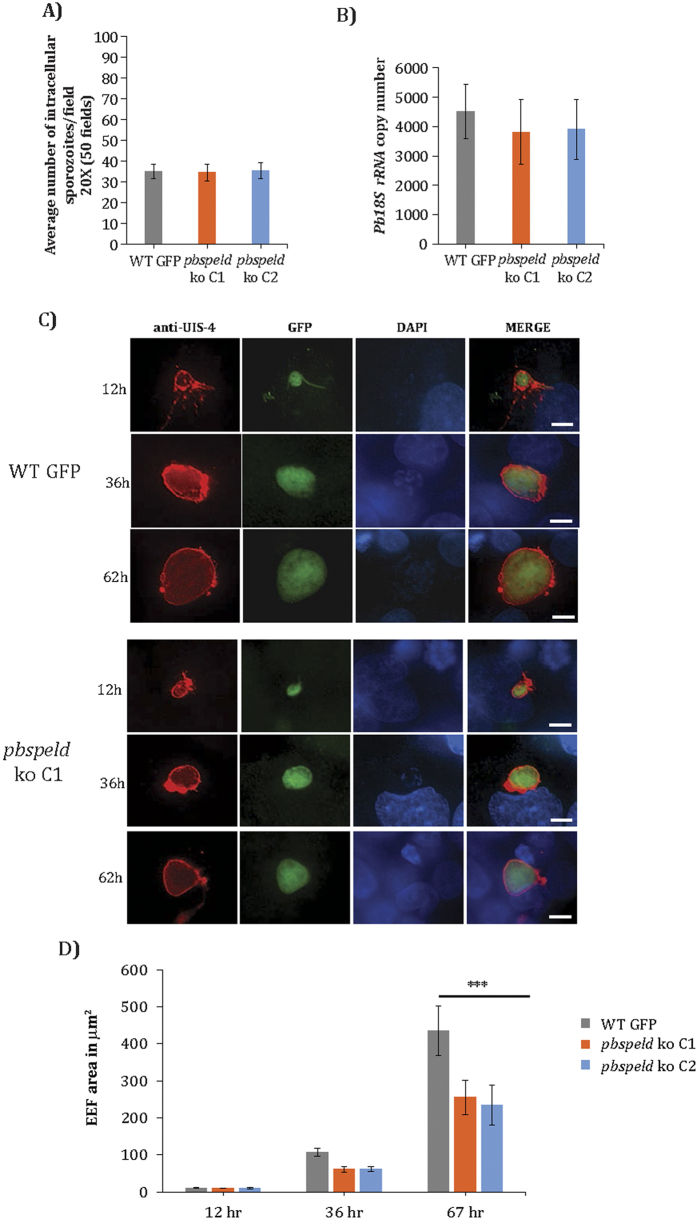
The Exo-erythrocytic forms (EEFs) of *pbspeld* ko show arrested growth at mid liver stage development. (**A**) Quantification of sporozoite infectivity by inside out assay in WT GFP, *pbspeld* ko clone C1 and C2. (**B**) Quantification of parasite burden in liver of C57BL/6 mice following intravenous injection of 10^4^ sporozoites of WT GFP, *pbspeld* ko clone C1 and C2 (n = 3 per group). (**C**) *In vitro* EEF development of WT GFP, *pbspeld* ko clone C1 and C2. Sporozoites were added to HepG2 cultures, and the development of EEFs was monitored at indicated post-invasion time points by IFA using UIS4 antibody specific for the parasitophorous vacuolar membrane (PVM) and DAPI that facilitated visualizing host and parasite nuclei. (**D**) Measurement of EEF area in WT GFP, *pbspeld* ko clone C1 and C2 at 12 h, 36 h and 62 h. Both *pbspeld* ko clone C1 and C2 show growth attenuation at 36 and 67 h (n = 15, ***p < 0.0005). Scale bar 10 μm.

The absence of blood stage infection in C57BL/6 mice exposed to mosquito bite or injection of *pbspeld* ko or an occasional break through infection in some mice implied either a compromised invasion of sporozoites into hepatocytes or inability of the invaded sporozoites to complete EEF development. To unravel which of these processes were affected in *pbspeld* mutants, we investigated the sporozoite infectivity both under *in vitro* and *in vivo* conditions. We first analysed the ability of sporozoites to glide under *in vitro* conditions (Supplementary Fig. S2A). We observed that *pbspeld* ko sporozoites were able to perform gliding motility that was indistinguishable from WT sporozoites. Next, we added 2 × 10^4^ sporozoites of clone 1 or clone 2 to HepG2 cells and compared their rates of infection to that of WT GFP sporozoites. Two hours post infection the cells were fixed and a differential inside out staining of sporozoites was performed to quantify the number of intracellular sporozoites^30^. We observed that both clones C1 and C2 invaded with similar efficiency and the number of intracellular sporozoites were comparable to WT GFP sporozoites (Fig. 3A). We next introduced 1 × 10^4^ sporozoites of clones C1 and C2 or WT GFP intravenously into mouse (3 mouse/group) and isolated livers at 15 hours post infection. Total RNA was obtained from infected livers and cDNA was synthesized. The cDNA was used for quantification of the *Pb18SrRNA* burden by qRT PCR. We noted almost similar parasite loads in liver infected with C1, C2 and WT GFP (Fig. 3B). We conclude that *pbspeld* depletion did not compromise hepatocyte trophism of the sporozoites and mutants behaved indistinguishably as WT with regards to commitment to hepatocyte infection. We next investigated the growth of both clones in HepG2 cells during different time points. HepG2 cells infected with both the mutant clones and WT sporozoites were fixed at indicated time points and stained with UIS4 antibody. The host and parasite nuclei were stained with DAPI. Both clones showed normal pattern of intracellular differentiation into EEFs at 12 hrs post infection of HepG2 cells and were similar in size to WT EEFs. At 36 hours the EEFs from both clones were smaller than WT, and the difference in size was more pronounced at 62 hrs time point (Fig. 3C) that revealed a distinct block in EEF development likely around 36 hr. The size of EEFs at each time point (n = 15) was measured for both clones as compared to WT GFP (Fig. 3D). Taken together, our results provide evidence for the role of *pbspeld* in maturation of EEFs.

### Generation of *pbspeldmCherry* transgenic line

**Figure 4:**
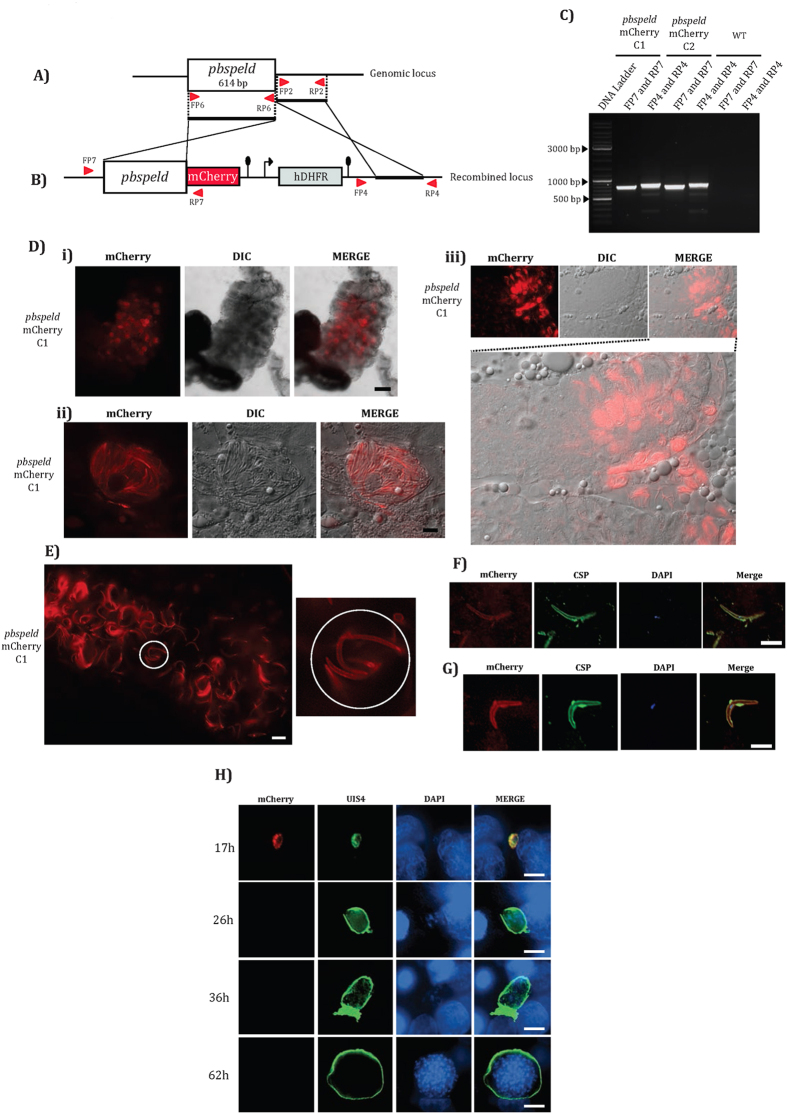
Generation of *pbspeldmCherry* transgenic line and demonstration of reporter expression in midgut sporozoites, salivary gland sporozoites and in early developing EEFs. (**A**) Genomic locus of *pbspeld* showing ORF, 3′ region and position of the primers. (**B**) Cloning of ORF (without stop codon) and 3′ region in the targeting vector pBC-mCherry-hDHFR. The *pbspeld* ORF was PCR amplified using primer set FP6/RP6 and cloned into ApaI/XhoI site of the targeting vector. The 3′ region of *pbspeld* was PCR amplified using primer set FP2/RP2 and cloned in the NotI/AscI site of the targeting vector. (**C**) Agarose gel showing products of diagnostic PCR with primer set FP7/RP7 and primer set FP8/RP8 indicating correct integration respectively in *pbspeldmCherry* transgenic clones C1 and C2. No product was amplified from genomic DNA of WT parasites using same set of primers. (**D**) *pbspeldmCherry* transgenic parasites express the reporter in oocyst and salivary gland sporozoite stages. (i) Dissected mosquito midgut showing oocyst expressing mCherry. Scale bar 200 μm. (ii) Higher magnification (100X) of oocyst showing mCherry expression in sporozoites within oocyst. Scale bar 10 μm. (iii) Dissected salivary gland lobe showing sporozoites expressing mCherry. Scale bar 10 μm. (**E**) Circles with white outline indicate sporozoites showing SPELD mCherry localization to the plasma membrane with in dissected salivary gland lobes. Scale bar 5 μm. (**F**) and (**G**) Colocalisation of mCherry with CSP in midgut sporozoites and salivary gland sporozoites respectively. The sporozoites were incubated with 3D11, a monoclonal antibody that recognizes the repeats of *P. berghei* CSP protein. Scale bar 10 μm. (**H**) *pbspeldmCherry* transgenic express reporter only at 17 h EEF development. The development of EEFs was monitored at indicated post-invasion time points by IFA using UIS4 antibody specific for the parasitophorous vacuolar membrane (PVM) and DAPI that facilitated visualizing host and parasite nuclei. Scale bar 10 μm.

To study the expression and localization of the product encoded by *pbspeld* we generated mCherry transgenic line. The organization of the *pbspeld* locus is shown in Fig. 4A. To generate the targeting construct, the *pbspeld* ORF and 3′ UTR were amplified using primer sets FP6/RP6 and FP2/RP2 and the PCR products were cloned into pBC-mCherry-hDHFR vector using ApaI/XhoI and NotI/AscI restriction sites respectively. The targeting construct was released following digestion with ApaI and AscI and electroporated into schizonts. Following successful DCO, *mCherry* cassette is placed in frame with the *pbspeld* locus (Fig. 4B). The transfectants were subjected to limiting dilution and two clones- C1 and C2 from independent transfections were confirmed for correct integration using primer sets FP7/RP7 and FP8/RP8 that amplified respectively 771 bp and 819 bp products only in *pbspeldmCherry* transgenic and not from WT parasites (Fig. 4C).

### *pbspeldm* *Cherry* transgenics express reporter in oocyst sporozoite, salivary gland sporozoite and in early EEF stage

To study the mCherry expression and localization of the product encoded by *pbspeldmCherry*, one of the cloned transgenic lines C1 was infected to mouse and analyzed for the reporter expression by live fluorescence microscopy. We did not detect mCherry expression in the asexual stages, gametocyte stages and at stages 26–30 hrs post fertilization in mosquito midguts suggesting that *pbspeld* promotor was not active at these respective stages. However, at day 14, midgut oocysts (Fig. 4D i), sporozoites within oocyst (Fig. 4D ii) and at day 18–21, the salivary glands sporozoites (Fig. 4D iii) were all positive for reporter expression. Deconvoluted images of intact salivary glands harbouring sporozoites revealed the localization of mCherry to the sporozoite plasma membrane (Fig. 4E). The membrane associated mCherry colocalised with CSP in both midgut (Fig. 4F) and salivary gland sporozoites (Fig. 4G). We also observed the localization of mCherry to the membrane of developing EEF at 17 hrs (Fig. 4H) that colocalised with UIS4, a parasitophorous vacuolar membrane protein. However, no mcherry expression was associated with EEFs at 26, 36 and 62 hours post infection.

### Immunization with *pbspeld* ko sporozoites generates pre-erythrocytic immunity

Since *pbspeld* ko sporozoites experience a block in mid liver stage development, we next tested the ability of the mutant sporozoites to mount a pre-erythrocytic immunity. Towards this end, we immunized C57BL/6 mice two times with 2 × 10^4^ *pbspeld* ko sporozoites within an interval of 2 weeks. Ten days after final immunization, all mice were challenged with 2 × 10^4^ WT sporozoites. Analysis of prepatent period in three independent experiments from clone 1 and 2 independent experiments from clone 2 revealed that nearly 50% of the immunized mice were protected from challenge (Supplementary Table 1). We conclude that a prime boost immunization regimen with *pbspeld* ko sporozoites induces immunity whose efficacy is nearly 50%. We next investigated if anti-sporozoites antibodies contributed to protection. We performed sporozoite IFA using pooled immune sera (obtained from mouse immunized with clone 1 and 2) that showed positive immunoreactivity in the range of 1:1600-1:3200 (Supplementary Fig. S2B, C and D). Further to test the sporozoite neutralizing ability of the immune sera, C57BL/6 mice were challenged with WT sporozoites incubated in immune sera. A significant delay in prepatent period that ranged from day 5.5 to day 6 was observed in mice that received sporozoites exposed to immune sera as compared to day 3.5 to 4 observed for mice that received sporozoites incubated in pre-immune sera (Supplementary Table 2). We implicate this delay in prepatent period to the ability of immune sera to partially neutralize the infectivity of sporozoites prior to hepatocyte invasion. Further, *in vitro* neutralization of WT *P. berghei* ANKA sporozoites in immune sera prior to its addition in HepG2 cells showed nearly 2 fold reduction in the EEF burden as measured by quantification of *Pb18SrRNA* copy number (Supplementary Fig. S2E). Our observations that *pbspeld* ko sporozoites glide in a similar manner as WT may explain the ability of these sporozoites to generate humoral response against secretory antigens (Supplementary Fig. S2A). Our results implicate that *pbspeld* mutant sporozoites induce protection through generation of partially protective anti-sporozoite antibodies.

### Microarray of *pbspeld* ko liver stage revealed dramatic changes in the global gene expression

**Figure 5:**
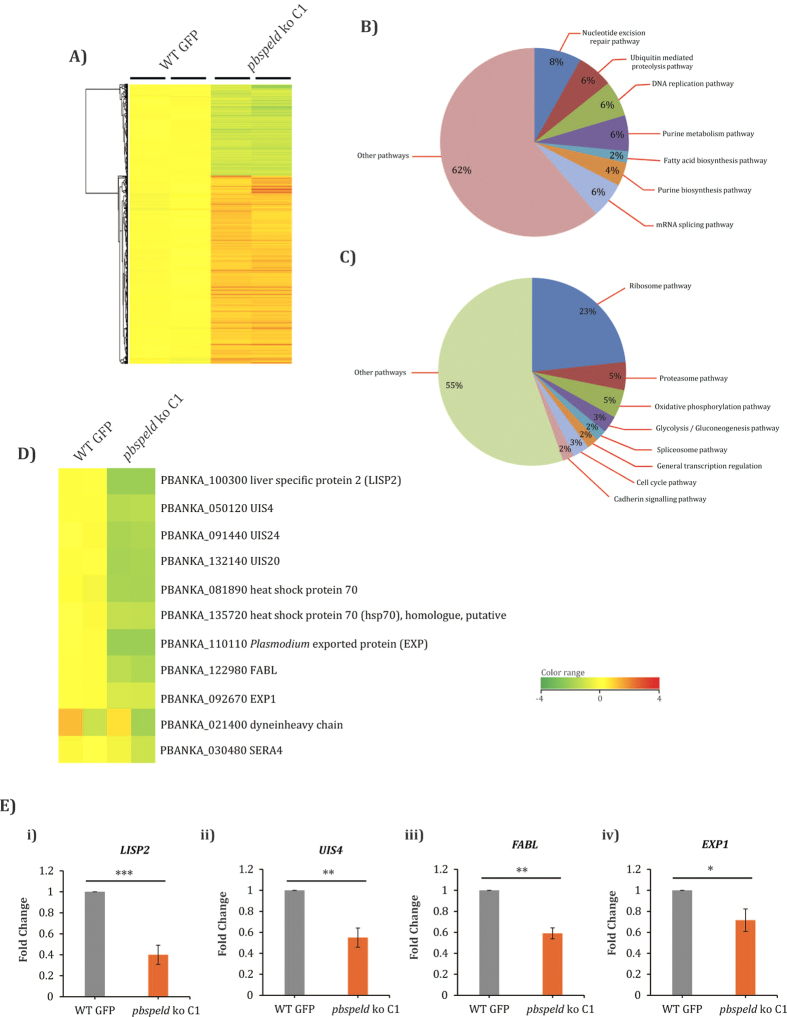
Microarray of 36 hrs liver stages of WT and *pbspeld* ko. (**A**) Heat map showing global gene expression changes in *P. berghei* liver stages at 36 hrs from WT and *pbspeld* ko. B) Pie diagram indicating the major functional pathways upregulated (**B**) and downregulated (**C**) in *pbspeld* ko. (**D**) Few of the well characterised liver stage specific genes downregulated in *pbspeld* ko. The plasmoDB ID of each of these genes with their probable functions are indicated. (**E**) Quantitative real time PCR showing downregulation of transcripts: *LISP2, UIS4, FABL* and *EXP1* in *pbspeld* ko. (*p < 0.05,**p < 0.005, ***p < 0.0005 as compared to WT).

Growth arrest of *pbspeld* ko parasites in mid liver stage and its inability to initiate a timely blood stage infection led us to analyze the global gene expression in the mutants. Microarray revealed major changes in the gene expression of *pbspeld* ko EEFs that showed upregulation of 1201 genes and downregulation of 552 genes (Fig. 5A). The important functional pathways up and down-regulated are indicated in pie diagram (Fig. 5B and C). The functional clusters that were upregulated belonged to nucleotide excision repair, ubiquitin mediated proteolysis, DNA replication, fatty acid synthesis, purine metabolism and mRNA splicing (Supplementary Fig. S3A). The important clusters that were downregulated belonged to general transcription by RNA pol 1, glycolysis/gluconeogenesis, spliceosome pathway and ribosomal genes (Supplementary Fig. S3B), cadherin signaling pathway, cell cycle pathway, proteosome pathway and oxidative phosphorylation pathway (Supplementary Fig. S3C). Consistent with the attenuation of the *pbspeld* ko during mid liver stage, several liver stage specific transcripts like *UIS4, LISP2, EXP1, FABL* and *SERA4* were significantly downregulated (Fig. 5D). The down regulation of few selected genes like *LISP2, UIS4, FABL* and *EXP1* were further validated by qRT-PCR (Fig. 5E). Amongst other downregulated genes were the putative orthologues of *P. yoelli* that were shown to be induced at different time points (24 hrs, 40 hrs and 50 hrs) in the transcriptomic studies of the *in vivo* late liver stages^31^. These included enolase (*PBANKA_121430*), receptor activated c-kinase (RACK) (*PBANKA_070390*), protein disulfide isomerase (*PBANKA_070280*), endoplasmin (GRP94) (*PBANKA_142730*), peptidyl-prolylcis-trans isomerase (*PBANKA_121650*), 1-cys peroxiredoxin (*PBANKA_122800*), proteosome subunit alpha type 6 (*PBANKA_122310*), biotin carboxylase subunit of acetyl CoA carboxylase (*PBANKA_133280*) and heat shock protein 110 (*PBANKA_121930*) (Supplementary Fig. S3D). The complete microarray data set is accessible from the NCBI GEO database (accession GSE72399) and GEO accession at: http://www.ncbi.nlm.nih.gov/geo/query/acc.cgi?acc=GSE72399. The differential changes in the gene expression of *pbspeld* mutants as observed in the microarray analysis were further validated by performing qRT-PCR. Four genes each selected from upregulated and down regulated category showed similar trend in qRT-PCR expression analysis (Supplementary Fig. S4A and Fig. 4SB). The global changes in the gene expression of several functional clusters of likely reflects the compromised EEF development in *pbspeld* mutants.

## Discussion

Malaria transmission to vertebrates occurs when female *Anopheles* mosquitoes introduces sporozoites into the skin of the vertebrate host while probing for a blood meal^2^. Following successful invasion of sporozoites into hepatocytes, they transform into EEFs inside a parasitophorous vacuole, a membrane bound structure partly derived from host plasma membrane and secretory proteins released by the invading sporozoites^32^. While the pre-erythrocytic stages are clinically silent, they however lead to onset of blood stage infections that are associated with all pathological manifestations of malaria. Therefore, blocking sporozoite entry into hepatocytes or transformation of sporozoites into EEFs in order to preclude blood stage infection has been an active area of research. Towards this end, deciphering the function of hypothetical genes highly upregulated in sporozoite stages have been rigorously pursued in recent past leading to understanding of several aspects of the *Plasmodium* pre-erythrocytic biology. Arguably, this pursuit has led to creation of single^22^,^33^ double^34^ and triply attenuated parasites^35^ whose efficacy as a whole organism pre-erythrocytic vaccines have been successfully proved. In the current study, we investigated the role of *PBANKA_191090* in pre-erythrocytic stages owing to its high transcription in salivary gland as determined through SAGE analysis^13^ and show that it is essential for maturation of EEFs.

The sporozoite membrane localization of PbSPELD is consistent with our *in silico* analysis that predicted transmembrane domain in its amino acid sequence. Our observations also concur with the proteomic analysis of sporozoite surface proteins that detected the orthologues of PbSPELD in both *P. yoelli* [Py: PY02432] and *P. falciparum* sporozoites (Pf: PF11_0545)^36^,^37^. Consistent with these results, PbSPELD colocalised with CSP, a major sporozoite surface protein^38^. However, unlike the known role of CSP in commitment of sporozoites to hepatocytes, PbSPELD was not required for invasion to hepatocyte as the mutant sporozoites invaded and initiated transformation normally both in the HepG2 cells and in liver hepatocytes. Interestingly the ko manifested a phenotype at a time point beyond supposed expression of the protein. It is only speculative that the mid liver stage development block may be associated in a yet unknown manner to protein function in sporozoite stages. Thus the early expression of PbSPELD in sporulating oocyst and salivary gland sporozoites is likely needed only to facilitate the sporozoite maturation within hepatocytes rather than its role in invasion of salivary glands or hepatocytes.

Much of global changes observed in transcriptome of *pbspeld* ko may not be a direct consequence of its depletion, but rather a secondary or indirect effect, induced by the lack of EEF maturation in the absence of PbSPELD. These changes likely reflected the steady state levels of transcripts at the time point that corresponded to the growth arrest. Downregulation of genes central to ribosome functions, general transcription by RNA polymerase 1, glycolysis/gluconeogenesis, cell cycle pathway, oxidative phosphorylation, proteasome in *pbspeld* ko may be a consequence of premature termination of the liver stages prior to completion of schizogony. Many gene products like *UIS4*^23^, *LISP2*^39^, *EXP1*^40^, *FABL*^41^ and *SERA4*^42^ are essential for maintenance of EEF as it progressively matures and down regulation of their expression provided a solid evidence for attenuation of EEFs in *pbspeld* mutants. The upregulation of certain genes clusters mediating mRNA splicing pathway, ubiquitin mediated proteolysis, nucleotide excision repair, DNA repair, purine metabolism pathway as observed in our microarrays analysis may be a consequence of the indirect effects of PbSPELD depletion and merits further investigation.

Several lines of evidence indicate that sporozoites attenuated either by irradiation or by genetic means induce protective immune responses^43^. The immune effectors involved are gamma interferons, antibodies and T cells^44^,^45^ and protective immunity generated is independent of the mode of attenuation^46^. The ability of the liver arrested parasites to induce protective CD8+ T cell immunity is reliant on exported antigens capable of activating T cell response^47^. Alternatively, cross presentation of antigens by liver resident professional antigen presenting cells have been shown following acquisition of infected hepatocytes harbouring aborted *Plasmodium* EEFs^48^. The complete protection associated with 50% of immunised mice upon challenge with WT sporozoites was indicative of powerful T cell response, and the relative contribution of different T cell subsets to this protection is currently under investigation. Additionally, the presence of anti-sporozoite neutralizing antibodies that partially block the infectivity of sporozoites coincided with the reactivity of the immune sera on WT sporozoite surface. Taken together, these observations point to the ability of *pbspeld* mutants in generating pre-erythrocytic immunity.

The extreme recalcitrance of *pbspeld* ko to complete liver stage schizogony even under high dose of sporozoite inoculation implicates that *Pb*SPELD depletion leads to high degree of liver stage attenuation. While merosome formation remains as the predominant mode of merozoite release from hepatocytes harbouring mature EEFs^49^, Baer *et al*., observed an alternative method of merozoite release from EEFs undergoing decay^50^. This phenomenon was recorded as early as 42 h after sporozoite infection, hours before merozoite differentiation begins and was regardless of EEF maturity and associated with the abortive liver stage development. While, SPELD showed a mid liver stage block in development, nonetheless, at 36 h time point we still detected limited schizogony. We propose that the abortive EEFs that under went few rounds of nuclear division may still release merozoites that may lead to occasional break through infection. The fact that this mode of merozoite release was also a rare event in *pbspeld* ko line is evident from the delay in the prepatent period to 9 days.

In conclusion, we propose *pbspeld* locus as a vulnerable target for malaria intervention. Based on sporozoite surface localization data, further studies are warranted if it can be a target of neutralizing antibodies. The association of PbSPELD on PVM during early EEF development and its absolute requirement in EEF maturation may suggest a speculative role of PbSPELD in nutrient acquisition and protein export^51^,^52^. Inability of these mutants to complete EEF development may likely be a consequence of one or more of these processes being compromised and merits further investigation. Having orthologues in other human species of *Plasmodium*^36^,^53^, it will be interesting to investigate if *speld* can be an additional candidate to achieve attenuation of human malaria liver stage parasites through targeted gene disruption.

## Methods

### Ethics Statement

Animal experiments were approved by the Institutional Animal Ethics Committee at University of Hyderabad (approval no: UH/SLS/IAEC/2014-1/9b and UH/SLS/IAEC/2014-1/9c.) and CSIR-Central Drug Research Institute (approval no: IAEC/2013/83), India. Maintenance and care of animals was in accordance with guidelines of the Committee for the Purpose of Control and Supervision of Experiments on Animals (CPCSEA), Government of India.

### RNA isolation from different stages of *P. berghei* ANKA and cDNA synthesis

Total RNA was isolated from different stages of the parasite using RNA isolation kit (Life Technologies) following manufacturer’s instructions. For cDNA synthesis, 2 μg of RNA was reverse transcribed in a reaction mixture containing 1X PCR buffer, 2.5 mM dNTPs, 5 mM MgCl_2_, 1.5 units RNAse inhibitor, 2.5 mM random hexamers and 1.5 units reverse transcriptase (Applied Biosystem). *pbspeld* transcript levels were quantified by real time PCR using primer pairs RT FP and RT RP. *Pb18SrRNA* was used as internal control to normalize the gene expression data^54^,^55^.

### Construction of the *pbspeld* (*PBANKA_091090*) ko targeting vector

For successful replacement *pbspeld* locus, a 586 bp of 5′ and 568 bp of 3′ fragments that flanked the target gene were amplified and cloned into the targeting vector pBC-GFP-hDHFR at XhoI/ClaI and NotI/AscI sites respectively. Amplification of the 5′ and 3′ fragment was done using primers FP1-RP1 and FP2-RP2 respectively. Vector backbone was removed from the targeting cassette by digesting with XhoI/AscI.

### Transfection and confirmation of *pbspeld* ko by site-specific integration PCR

*P. berghei* transfections were done essentially as described earlier^56^. Two independent transfections (T1 and T2) were performed with the targeting construct. The transfected parasites were injected intravenously into mouse and following 24 hrs of injection, the mice were kept on pyrimethamine (Sigma. After selection, when the parasitemia reached around 5%, genomic DNA was isolated from transfected (T1 and T2) *P. berghei* blood stages using a genomic DNA isolation kit (Genetix), following manufacturer’s instructions. To confirm the site-specific integration of the *pbspeld* ko construct, PCR was performed using gDNA as a template. Diagnostic primers were designed, such that the forward primer FP3 flanked upstream of the 5′ recombined fragment and the reverse primer RP3 flanked within the GFP cassette. A second set of diagnostic primers were designed where the forward primer FP4 flanked within the hDHFR cassette and reverse primer RP4 flanked a sequence beyond the site of 3′ fragment integration. Following this confirmation, clonal lines of the ko was generated by process of parasite limiting dilution. Single clones were further confirmed by diagnostic PCR using forward primer FP5 and reverse primer RP5 designed within the target gene ORF.

### Transmission of *pbspeld* ko and *P. berghei* WT GFP parasites to *Anopheles* mosquitoes

Female *Anopheles* mosquitoes were allowed to obtain blood meal from mouse harbouring gametocytes of either WT GFP or *speld* ko line. The infected mosquito cage was placed in a chamber maintained at 20 °C and 80% RH for 18–22 days to facilitate the completion of sexual development and for the formation of salivary gland sporozoites. Successful transmission of parasites to mosquitoes was monitored by observing the oocysts on mosquito midguts on day 14. To obtain the salivary glands sporozoites, mosquitoes were dissected and salivary glands were isolated post day 18 of feeding. The glands were disrupted by crushing and the sporozoites numbers were enumerated using hemocytometer.

### Gliding motility assay

Sporozoites were added to Lab-Tek wells pre-coated with mAb 3D11 for 1 h at 37 °C and assay was performed as described earlier^57^. After incubation, medium was removed and wells were fixed and stained with biotinylated mAb 3D11 followed by streptavidin-FITC (Sigma) in order to visualize the CS protein-containing trails.

### Sporozoite invasion assay

HepG2 cells were seeded in eight-chambered Lab-Tek wells that were coated with collagen type 1 from Rat tail (BD Bioscience). Sporozoites (2 × 10^4^) were added per well and after 1 h at 37 °C, the cells were washed, fixed, and sporozoites were stained with a double-staining assay that distinguishes intracellular from extracellular sporozoites^30^.

### Quantitative determination of *in vivo* infectivity

Female C57BL/6 mice were infected by intravenous injection of 1 × 10^4^ sporozoites and parasite load in each mouse liver was determined 15 hrs post infection by real time PCR^58^. The primers used for amplification of *P. berghei* *18SrRNA* were Pb18SrRNA-F and Pb18SrRNA-R.

### *In vitro* EEF development and Immunofluorescence assay (IFA)

For obtaining the EEFs, the HepG2 cells were cultured in 24 well culture plates (Corning). When HepG2 cells reached 60–70% confluency, 2 × 10^4^ sporozoites were added per well. Cultures were fixed at different time points of EEF development and IFA was performed as described earlier^59^. Briefly, the cultures were incubated for 1 h with UIS4 antibody and immunoreactivity reveled by anti-rabbit antibody conjugated to Alexa Fluor 594 (Life Technologies). The HepG2 and parasite nuclei were stained with DAPI. After final wash the coverslips were mounted using antifade reagent (Life Technologies). To reveal the stage specific expression and localization of the reporter in the *pbspeldmCherry* transgenics, the oocyst and salivary gland sporozoites were fixed and stained with 3D11 monoclonal antibody^38^ which reacted with the CSP, a major surface protein of sporozoite. The CSP immunoreactivity was revealed by using antimouse antibody conjugated to Alexa Fluor 488. To monitor the expression of mCherry in the developing liver stages, 2 × 10^4^ *pbspeldmCherry* transgenic sporozoites were added to HepG2 cultures and harvested at different time points. All processed slides were visualised using a Nikon (Ni-E AR) upright fluorescent microscope. Images were procured using monochrome camera (Andor) and captured images were processed using software NIS elements AR.

### Generation of mCherry transgenic parasites for studying the localization of SPELD

A 614 bp of *pbspeld* ORF was amplified (without stop codon) using primer pair FP6-RP6. A 568 bp of 3′UTR of *pbspeld* was PCR amplified using primer pair FP2-RP2. Both *pbspeld* ORF and 3′ UTR were cloned into pBC-mCherry-hDHFR vector using ApaI/XhoI and NotI/AscI restriction sites respectively. The targeting construct was separated from vector back bone and transfection was performed as described in earlier section. After obtaining successful transfectants, 5′ and 3′ integration was confirmed by using primer pair FP7-RP7 and FP8-RP8 respectively. Primer sequences are given in Supplementary Table 3.

### Analysis of protective efficacy of *pbspeld* ko sporozoite immunisation

Six to eight weeks old C57BL/6 mice were immunized twice with 2 × 10^4^ *pbspeld* ko sporozoites. As an appropriate control, we immunized another group of mice (n = 6) with crushed lysates of uninfected salivary gland that served as mock immunized group. The duration between priming and boosting was 14 days. Ten days after boosting, a group of 6 immunised and naïve mice were challenged with 2 × 10^4^ *P. berghei* WT sporozoites. The prepatent period was determined by performing Giemsa staining of blood smears from day 3 post infection.

To determine sporozoite antibody titers, sera were collected from a group of 6 immunized mice prior to challenge. The sera were pooled and diluted in the range of 1:50 to 1:100,000. Wild type sporozoites obtained from dissected salivary glands were spotted on multiwell slides and incubated with different dilutions of pooled immune sera. Sporozoites were incubated in parallel, either with pre immune sera or 3D11 monoclonal antibody^38^ against CSP which were considered as negative and positive controls respectively. The sporozoite immunoreactivity was revealed using anti-mouse antibody conjugated to Alexa Fluor 488.

### Sporozoite neutralization assay

Sera obtained from mice that were twice immunized with *speld* ko sporozoites were pooled and used for performing sporozoite neutralization assays. In brief 6 × 10^4^ *P. berghei ANKA* WT sporozoites were incubated at room temperature in 50 μl of either pre-immune or immune sera. Following 35 minutes of incubation, the samples were diluted with incomplete DMEM and made up to 600 μl. Two groups of C57BL/6 mice (3/group) received 2 × 10^4^ sporozoites intravenously incubated either in pre immune or immune sera and their pre patent periods were monitored from day 3 post injections.

### Microarray of *pbspeld* ko EEFs

For microarray analysis of *speld* mutants, an Agilent Custom *Plasmodium berghei* gene expression microarray slide having 4 × 44k format designed by Genotypic Technology Private Limited was used that comprised of a total number of 43803 features including 5155 numbers of probes, and 1417 Agilent control features. The array covered 5106 number of transcripts that were sourced from Plasmodb database. The details of microarray hybridization, Scanning and data analysis is given in supplementary methods.

### Quantitative real time PCR (qRT-PCR) for validation of microarray data

To validate the expression of the genes up and down regulated in microarray, HepG2 cells were infected with WT and *pbspeld* ko sporozoites as described above and incubated for 36 hrs. For the gene copy number measurements RNA was isolated from cells and cDNA was made followed by quantitative PCR. The primer sequences of the genes that were validated by qRT-PCR is given in supplementary Table 4.

## Additional Information

**How to cite this article**: Al-Nihmi, F. M. A. *et al*. A Novel and Conserved *Plasmodium* Sporozoite Membrane Protein SPELD is Required for Maturation of Exo-erythrocytic Forms. *Sci. Rep.* **7**, 40407; doi: 10.1038/srep40407 (2017).

**Publisher's note:** Springer Nature remains neutral with regard to jurisdictional claims in published maps and institutional affiliations.

## Supplementary Material

Supporting Information

## Figures and Tables

**Table 1:** Transmission of *pbspeld* ko sporozoites through mosquito bite does not induce blood stage infection in mouse.

Parasite strain	Experiment number	Mice positive/mice exposed to mosquito bite	Pre patent period (days)
WT	I	3/3	3.5
	II	3/3	3.5
*pbspeld* ko C1	I	0/3	Negative until day 15
	II	0/3	Negative until day 15
	III	0/2	Negative until day 15
	IV	0/2	Negative until day 15
*pbspeld* ko C2	I	0/3	Negative until day 15
	II	0/3	Negative until day 15

All blood meal positive mosquitoes following bite experiment were dissected to collect salivary glands to confirm the presence of GFP expressing sporozoites under fluorescent microscope. Pre patent period: defined as time required for the appearance of blood stages following infection with sporozoites.

**Table 2:** High dose of *pbspeld* ko sporozoites delivered through intravenous (i.v) route induce an occasional break through infection in mice with delayed pre patent period.

Parasite strain	Experiment number	Mice positive/mice injected	Pre patent period (days)
WT	I	3/3	4
	II	3/3	4
*pbspeld* ko C1	I	1/4	9
	II	0/4	Negative until day 15
	III	0/3	Negative until day 15
	IV	0/2	Negative until day 15
*pbspeld* ko C2	I	0/3	Negative until day 15
	II	0/3	Negative until day 15
