# Supplementary material for: A Novel and Conserved Plasmodium Sporozoite Membrane Protein SPELD is Required for Maturation of Exo-erythrocytic Forms
Source: Sci Rep. 2017 Jan 9;7:40407. doi: 10.1038/srep40407 (PMC5220379; doi:10.1038/srep40407)
Supplement: Supporting Information [file srep40407-s1.pdf]

A Novel and Conserved *Plasmodium* Sporozoite Membrane Protein SPELD is Required for Maturation of Exo-erythrocytic Forms

Faisal Mohammed Abdul Al-Nihmi[1+], Surendra Kumar Kolli[1+], Segireddy Rameswara Reddy [1+], Babu S Mastan[1+], Jyothi Togiri[1], Mulaka Maruthi[1], Roshni Gupta[2], Puran Singh Sijwali[3], Satish Mishra[2*], Kota Arun Kumar[1*]

[1] Department of Animal Biology, School of Life Sciences, University of Hyderabad, Hyderabad 500046, India.

[2] Division of Parasitology, CSIR-Central Drug Research Institute, Lucknow 226031, India.

[3] CSIR-Centre for Cellular and Molecular Biology (CCMB), Habsiguda, Uppal Road, Hyderabad 500007, India.

*Corresponding authors S.M. (email: satish.mishra@cdri.res.in) or K.A.K. (email: kaksl@uohyd.ernet.in).

+These authors contributed equally to this work.

Supplementary Fig. S1

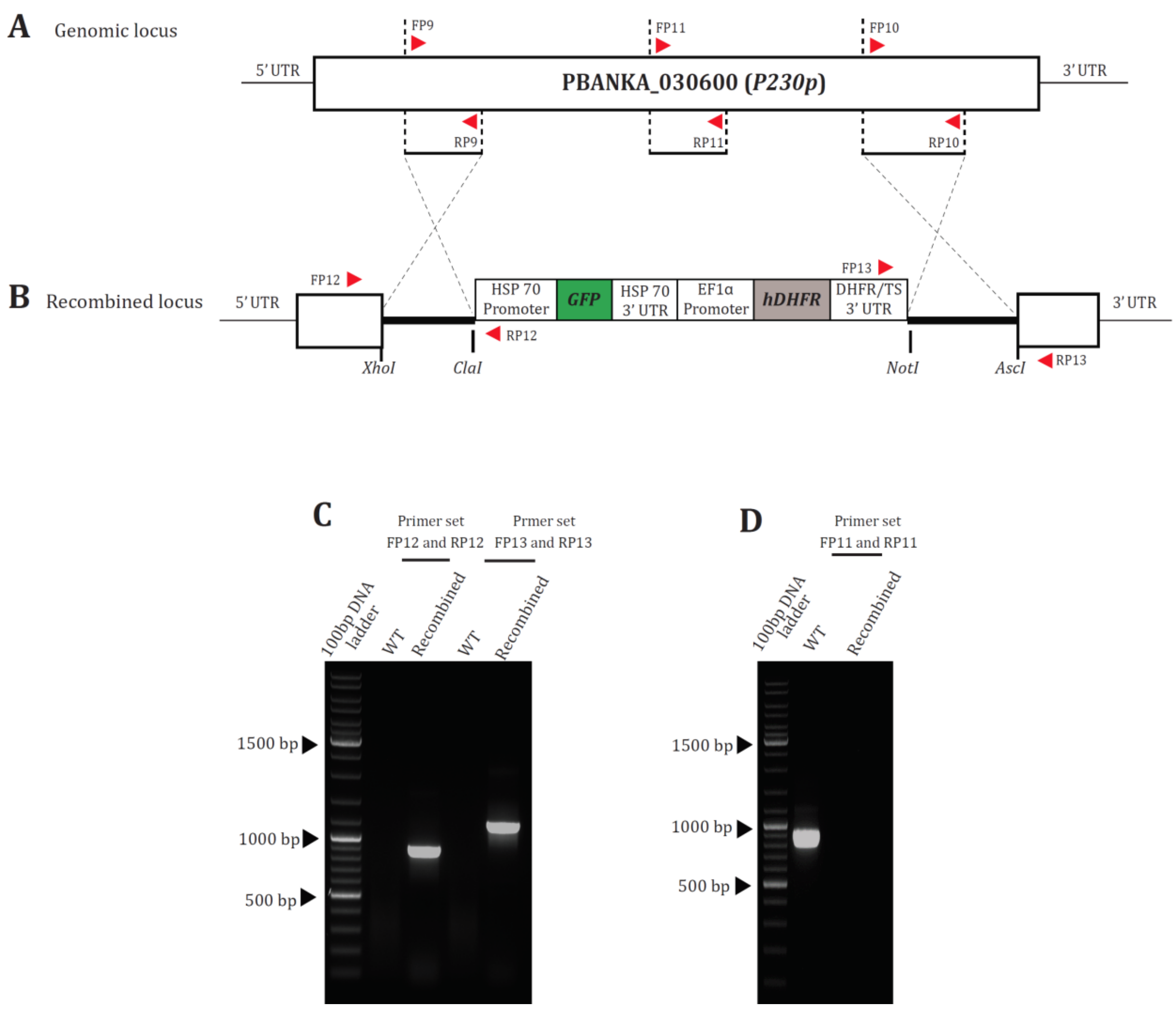

Supplementary Fig. S1: Generation of *Pb* ANKA WT GFP parasite line.

(A) Genomic locus of *P230p* (PBANKA_030600) locus showing the position of the primers used for amplification of fragment A and B within ORF of *P230p* and for confirming the integration of the cassette following DCO recombination. Fragment A and B were PCR amplified using primer sets FP9/RP9 and FP10/RP10 and was cloned respectively at XhoI/ClaI and NotI/AscI sites of the targeting vector. **(B)** Recombined locus formed after successful double crossover recombination at the *p230p* locus. **(C)** Agarose gel showing products of diagnostic PCR with primer sets FP12/RP12 and primer sets FP13/RP13 indicating correct integration at regions A and B respectively. **(D)** Agarose gel showing product amplified using primer set FP11/RP11 with in the *P230p* ORF from the genomic DNA of WT parasite but not from *P230p* transgenic locus line confirming successful clonal dilution of the transgenic line.

Supplementary Fig. S2

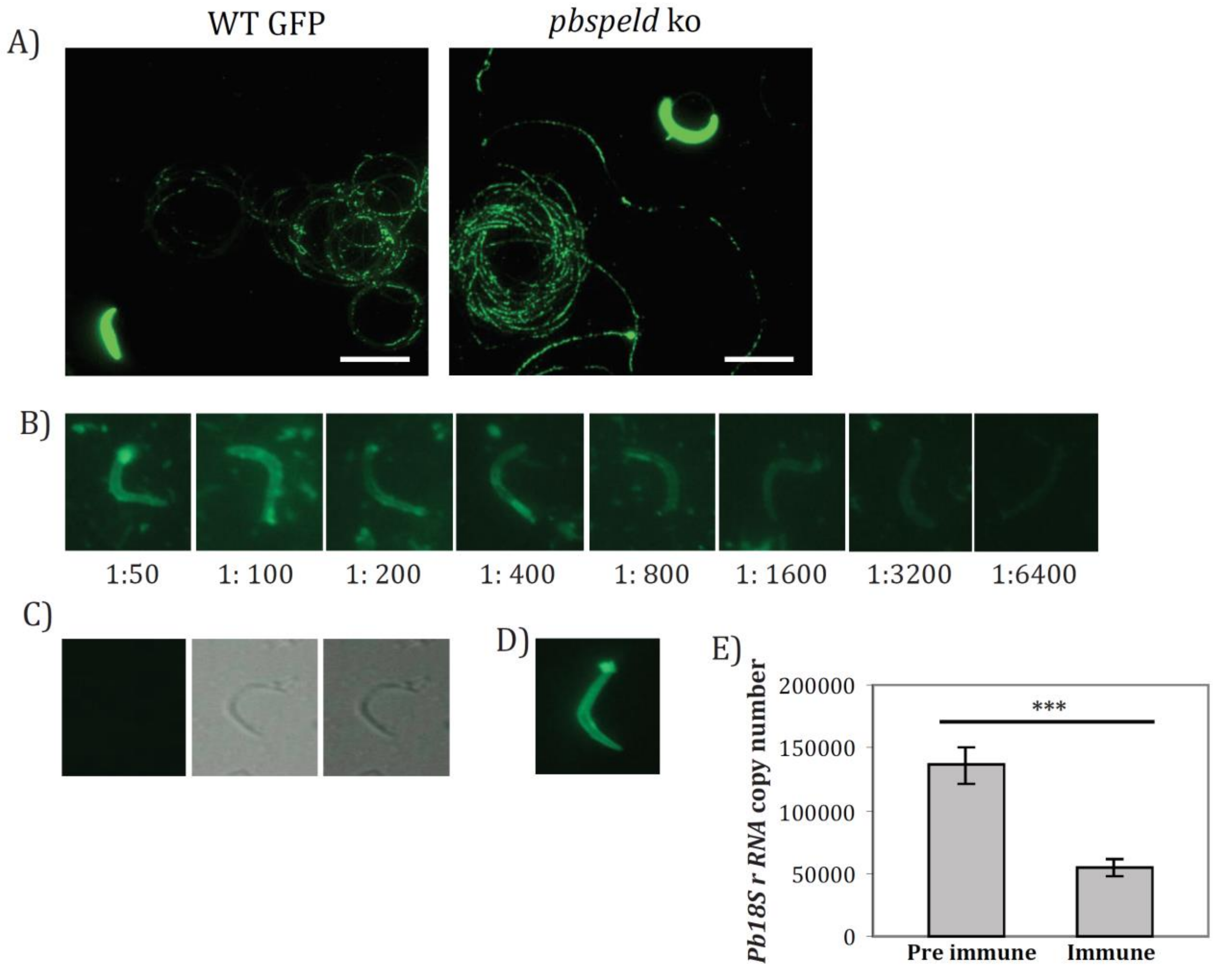

Supplementary Fig. S2: Release of sporozoite secretory antigens and reactivity of *pbspeld* ko sporozoite immune sera with WT sporozoites. A) Gliding motility of WT and *pbspeld* ko sporozoites showing release of CSP antigen as trails. Scale bar 10μm. B) Pooled immune sera obtained form 5 mice were subjected to a serial two fold dilutions in PBS. The dilutions of sera as indicated were incubated on fixed WT *P. berghei ANKA* sporozoites. The immuno-reactivity was revealed using an anti-mouse secondary antibody conjugated to Alexa Flour 488. Pre-immune sera (C) was used as a negative control and 3D11 monoclonal antibody (D) was used as a positive control. E) Analysis of *Pb18S rRNA* burden in HepG2 cells following addition of $2X10^4$ WT sporozoites following 35 min incubation in pre immune and immune sera. ***$p<0.0005$ compared to WT.

Supplementary Fig. S3A

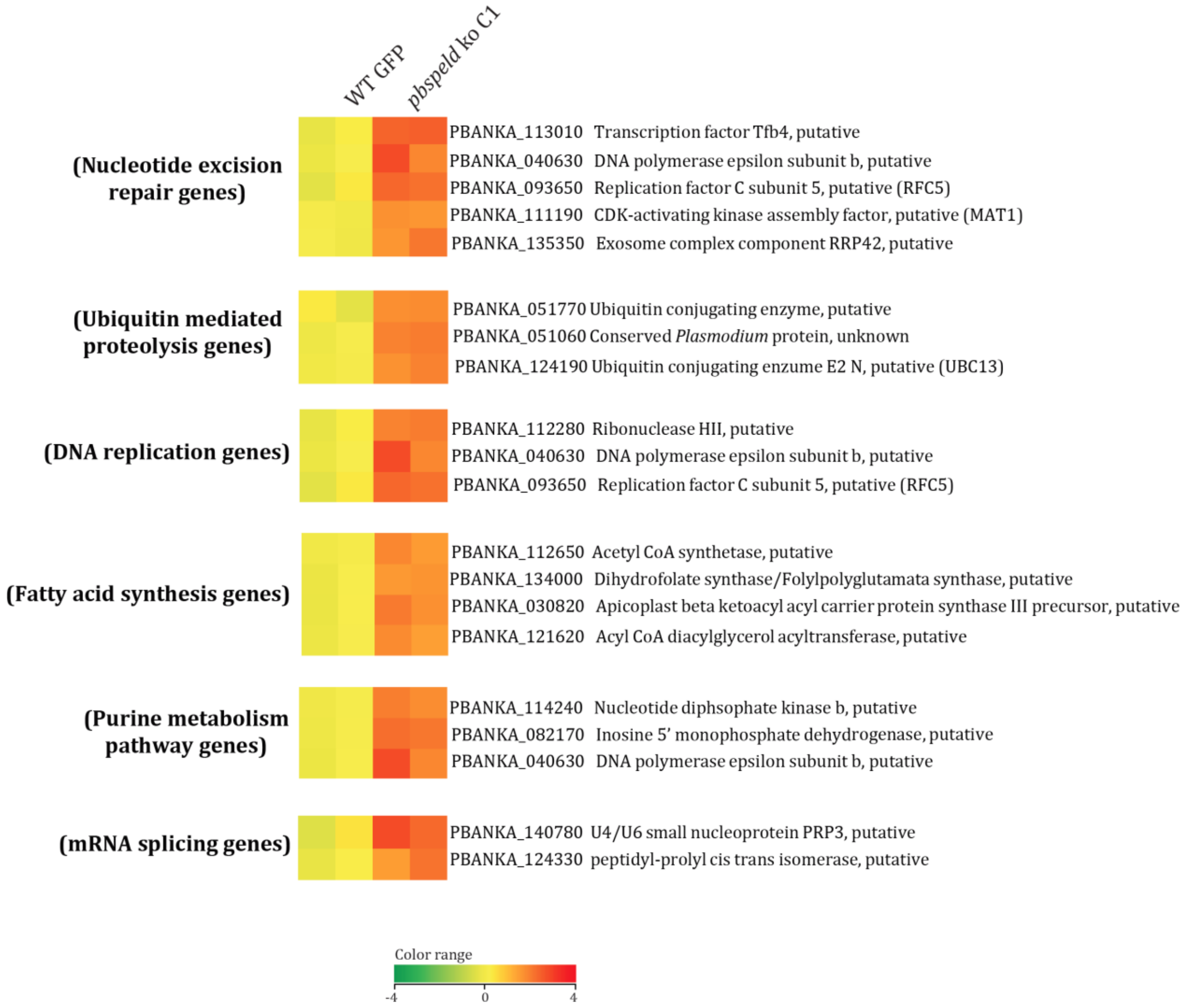

Supplementary Fig. S3A: Genes indicated in the functional clusters belonging to nucleotide excision repair, ubiquitin mediated proteolysis, DNA replication, fatty acid synthesis, purine metabolism and mRNA splicing are upregulated in *pbspeld* ko EEF's. The plasmoDB ID of each of these genes with their functions are indicated.

Supplementary Fig. S3B

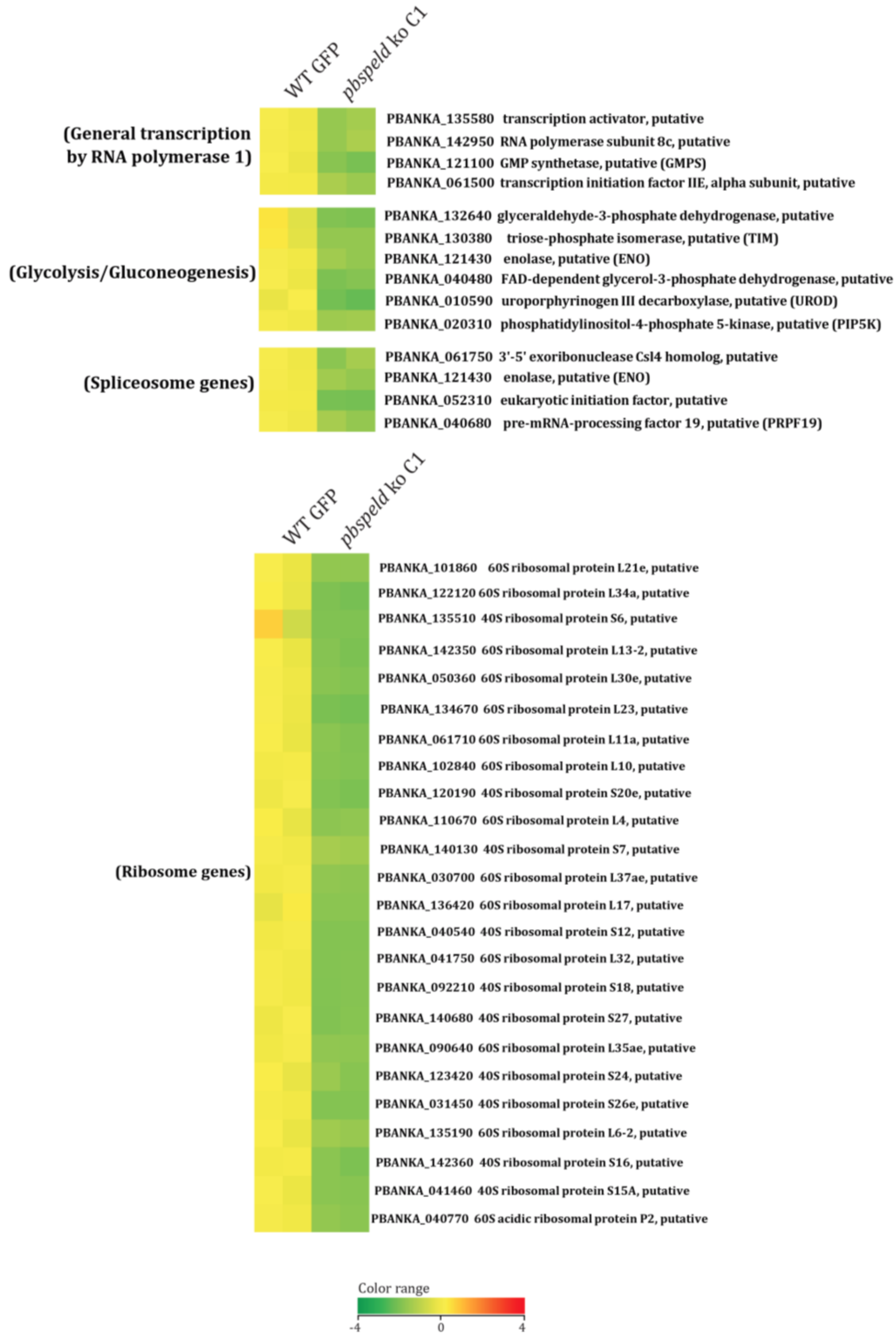

Supplementary Fig. S3B: Genes included in functional clusters belonging to general transcription by RNA pol 1, glycolysis and gluconeogenesis, splicesome and ribosome genes are downregulated in *pbspeld* ko EEF's. The plasmoDB ID of each of these genes with their functions are indicated.

Supplementary Fig. S3C

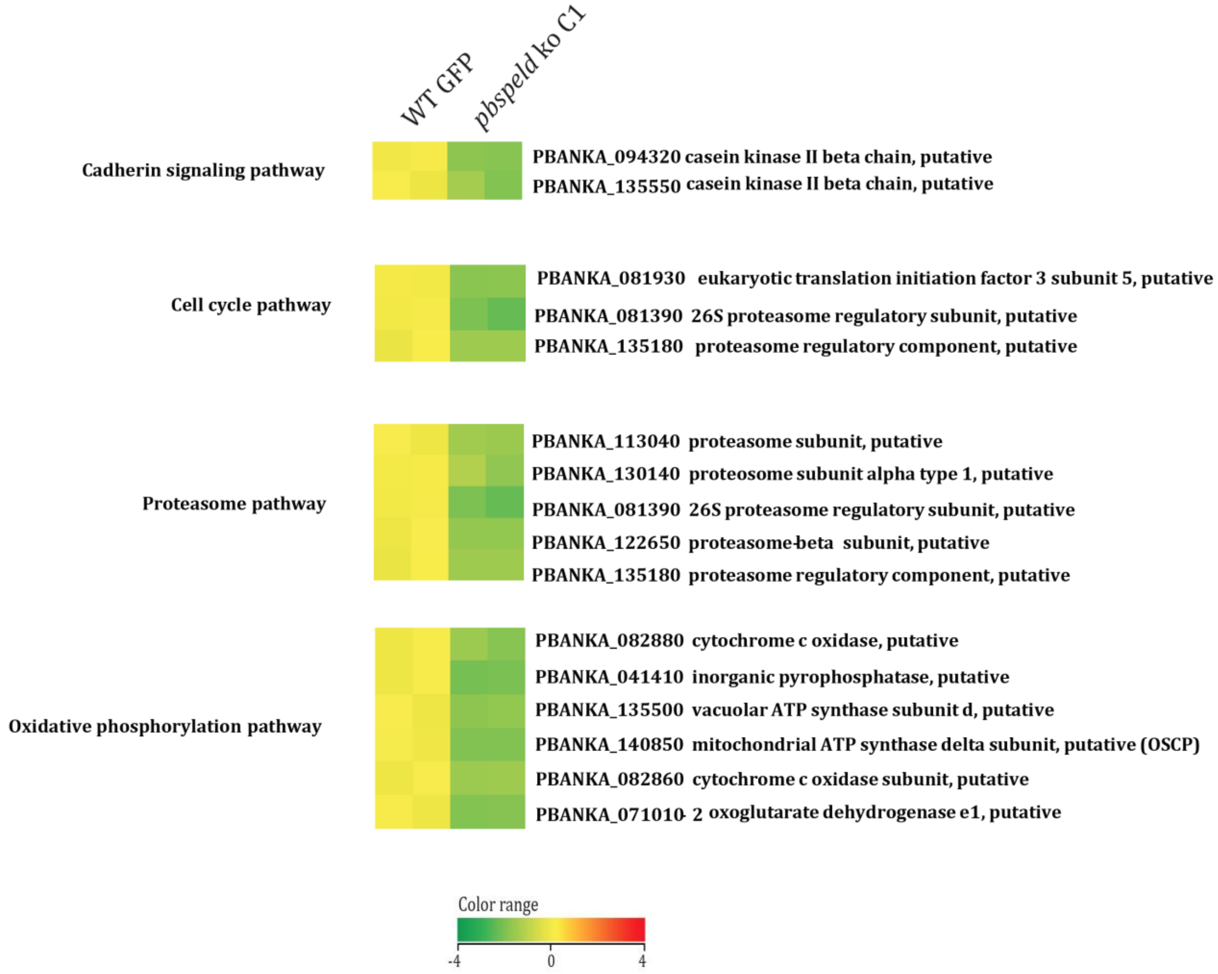

Supplementary Fig. S3C: Genes included in functional clusters belonging to cadherin signaling pathway, cell cycle pathway, proteosome pathway and oxidative phosphorylation pathway are downregulated.

Supplementary Fig. S3D

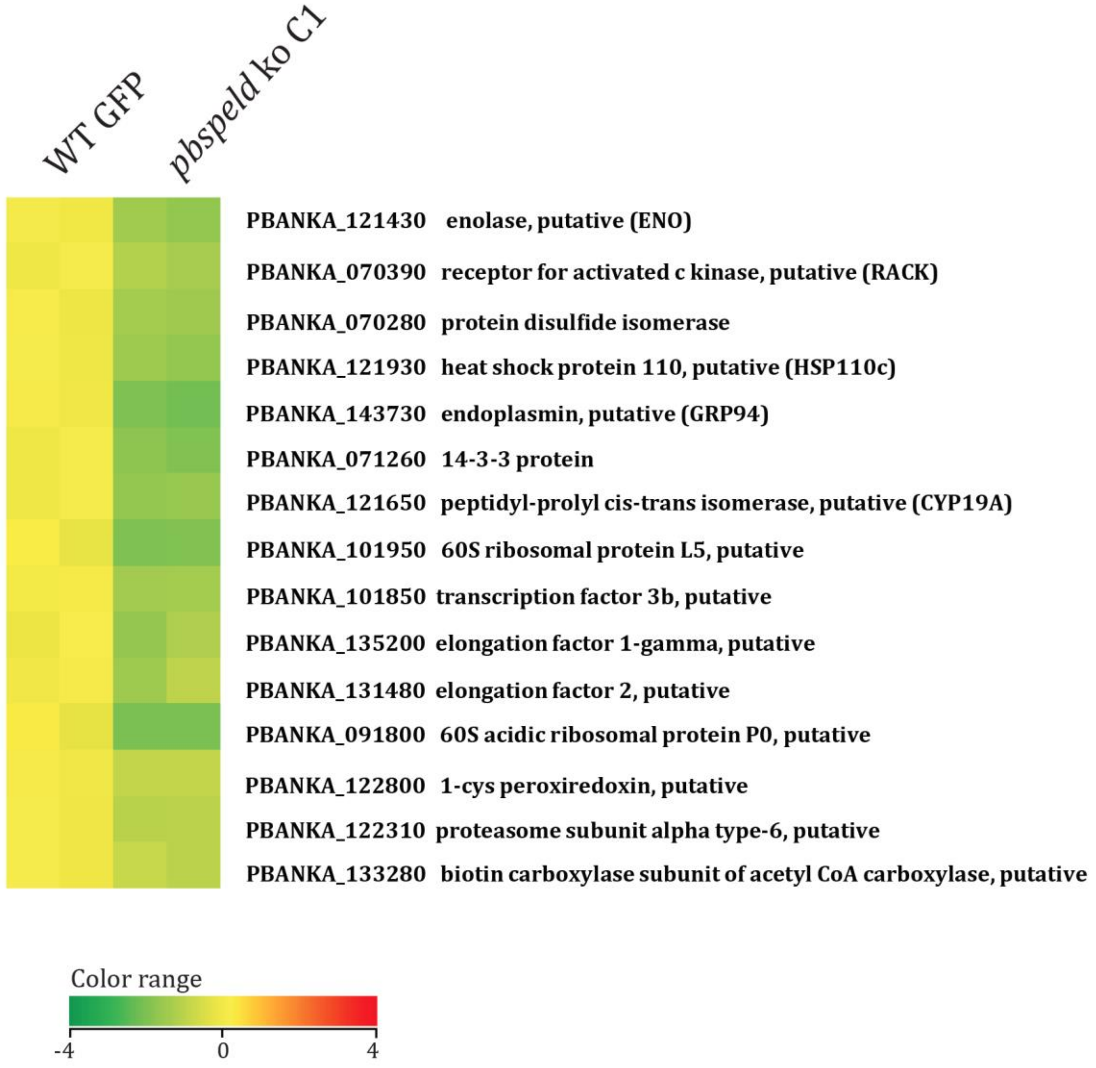

Supplementary Fig. S3D: List of putative genes that have been reported to expressed during 24 hrs to 50 hrs in the liver stages (Reference no. 31) were downregulated in *pbspeld* ko EEF's. The plasmoDB ID of each of these genes with their functions are indicated.

Supplementary Fig. S4A

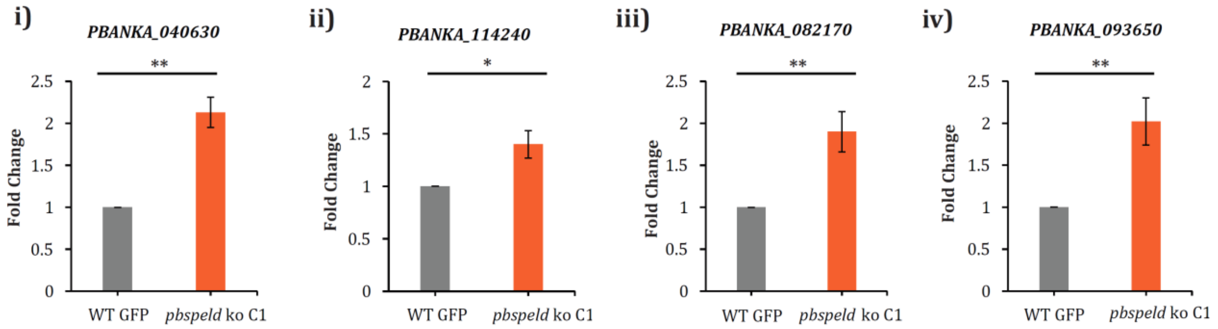

Supplementary Fig. S4A: Validating the expression of selected genes by qRT-PCR upregulated in microarray. cDNA was generated from HepG2 cultures infected with WT and *pbspeld* ko at 36h time point. The cDNA was used as template for analyzing the expression of indicated genes. **p value<0.005 compared to WT.

Supplementary Fig. S4B

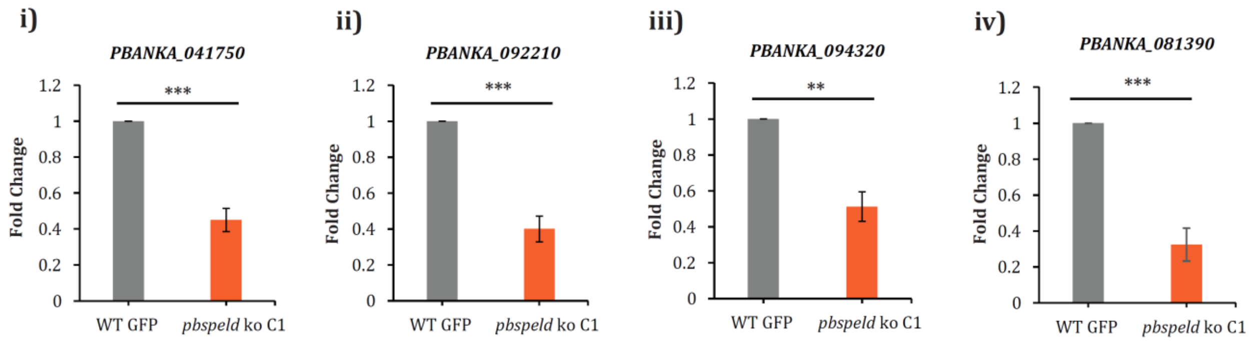

Supplementary Fig. S4B: Validating the expression of selected genes by qRT-PCR downregulated in microarray. cDNA was generated from HepG2 cultures infected with WT and *pbspeld* ko at 36h time point. The cDNA was used as template for analyzing the expression of indicated genes. ***p value<0.0005 and ** p value 0.005 compared to WT.

Supplementary Table 1.

A prime boost immunization regimen with $2X10^4$ *pbspeld* ko sporozoites induces protective immunity

**Immune status of C57BL/6 mice**	**Number of mice used for challenge with WT sporozoites**	**Challenge dose/mouse**	**Mice positive/mice injected**	**Pre patent period (days)**
Naive	3	$2X10^4$	3/3	4
Twice immunized with *pbspeld* ko sporozoites (Clone 1)	6	$2X10^4$	3/6	8/9
	5		2/5	
	7		3/7	
Twice immunized with *pbspeld* ko sporozoites (Clone 2)	6	$2X10^4$	2/6	8/9
	5		2/5	

Supplementary Table 2.

Analysis of pre patent period following incubation of WT *P. berghei* sporozoites in pre immune or *pbspeld* ko sporozoite immune sera

**Group**	**No. of C57BL/6 mice/group (3)**	**Pre patent period (days)**
WT *Pb ANKA* sporozoites incubated in pre-immune sera	Mice#1	4
	Mice#2	4
	Mice#3	4
WT *Pb ANKA* sporozoites incubated in pooled immune sera	Mice#1	6
	Mice#2	6
	Mice#3	5.5

Supplementary Table 3.

Primers used in this study

S. No.	Primer Name	Primer Sequence
1	RT FP	TATTTATTACCCTGCGGATA
2	RT RP	ATACTCAACGTGATATTTCCA
3	FP1	AGTCTCGAGATTATTAAACGTGAGGAATT
4	RP1	ACTATCGATAAAATGTGCTTAAACAATGA
5	FP2	ATAGCGGCCGCAAGCAAACAATAAACACTTA
6	RP2	GTAGGCGCGCCTGCATTATGAAACTGTCA
7	FP3	TGTCTATTTCTAATGTTCTTA
8	RP3	TTCCGCAATTTGTTGTACATA
9	FP4	GTTGTCTCTTCAATGATTCATAAATAG
10	RP4	ACCCAAACGAGACATATATA
11	FP5	ACTATTTATTACCCTGCG
12	RP5	TTAAGGATAAAATATAGCAGT
13	FP6	ATAGGGCCCATGACCAATCAAGTGTTAGA
14	RP6	AGTCTCGAGAGGATAAAATATAGCAGTAGG
15	FP7	TAACGTTATTTTATTTTTCTTGT
16	RP7	ACCTTGAAGCGCATGAACTCCTTGATGAT
17	FP8	GTTGTCTCTTCAATGATTCATAAATAG
18	RP8	ACCCAAACGAGACATATATA
19	Pb18SrRNA-F	AAGCATTAAATAAAGCGAATACATCCTTAC
20	Pb18SrRNA-R	GGAGATTGGTTTTGACGTTTATGT
21	FP9	AACTCGAGTCATGGATCATATCCACTAACAAT
22	RP9	AATATCGATTGTGTTTTATTTGGATGTGCAAT
23	FP10	ATGCGGCCGCTTCTCTTGAGCCCGTTAATGAA
24	RP10	ATGGCGCGCCTAGGAAATTTGTTTATTTTTAT
25	FP11	AGAATTGTATATGGTAAAGAACCTACTAACACAA
26	RP11	CCGCAATTTGTTGTACATAAAATAG
27	FP12	GTTGTCTCTTCAATGATTCATAAATAG
28	RP12	ATAGTGACTTTCAGTGAAATCGCAAACATAAG
29	FP13	ATTTCCAAAACCGTTAGAATATGTAGCATTACA
30	RP13	TTGGCGTCCCATCTATGCTACTCACTT

Supplementary Table 4.

Primers used for qRT PCR for validating up and down regulated genes in microarrays

S. No.	Primer Name	Primer Sequence
1	LISP2 RT FP	GCATTACTACTGAATCTTCATCGTGCACATT
2	LISP2 RT RP	GTATTTCCATTTGCGTCAATATCCTCAAG
3	UIS4 RT FP	GAACCCTGAAGTTCGAGAAAAATTTAGAATTGG
4	UIS4 RT RP	TCTGGTGAATTTTCTGGTGAATTTTCTGGTGA
5	FABL RT FP	GGAGTATGGCCCCCTGTTTATAACAT
6	FABL RT RP	TCCAGCATCAAGTGGTAAAACATCGA
7	EXP1 RT FP	CCAAAAATGTTATTAAAAAACCAGCAGAACCA
8	EXP1 RT RP	TGGCAAGGGCTACGTTTGCTTTTCTTAA
9	PBANKA_040630 RT FP	CAAGATATTTTATTTTTAGAAGATGAAACAGCACGA
10	PBANKA_040630 RT RP	ATATAACGAACCATTGCTCATACCAACAC
11	PBANKA_114240 RT FP	TAAGCAAGGAAGAAAATTAATTGGGGAAACAA
12	PBANKA_114240 RT RP	TGCTGAGGCAACTGAATCACTACCAT
13	PBANKA_082170 RT FP	AATTGGAATGGGAAGTGGTTCTATCTGT
14	PBANKA_082170 RT RP	TCCACCATCTGCTATTGTTTTTATATTTCGAG
15	PBANKA_093650 RT FP	AGCCGGATTAAGACGAACCTTAGAAA
16	PBANKA_093650 RT RP	CTTCTTCAGTTGGTAATGGAACTCTTATACA
17	PBANKA_041750 RT FP	TCCAATAGGTTTATGCGTGTTAAGCCT
18	PBANKA_041750 RT RP	GTGTCTTGTTTTCTTATTACTTCCATATCC
19	PBANKA_092210 RT FP	GAGAATTAACAACTGAAGAAATCAATAATATTGTTCA
20	PBANKA_092210 RT RP	TTAGCTATGACATGTAAATTCTTTCCATCCT
21	PBANKA_094320 RT FP	GAGATGCTCCTGATGATGAACATGC
22	PBANKA_094320 RT RP	GAGCAATGACCAAAGTCTCCCTTAATATA
23	PBANKA_081390 RT FP	GTGCACACAAATCAAACTATTTTCCCAGT
24	PBANKA_081390 RT RP	CATCTTCTGCTTCAAATGCACCTATTCTA

Supplementary Methods:

Retrieval of target genes sequences

Two public domain databases namely PlasmoDB (http://www.plasmodb.org/plasmo) and Sanger gene data base (http://www.genedb.org/Homepage/Pberghei) were used to retrieve the PBANKA_091090 gene sequence and its *Plasmodium* homologs and PBANKA_030600 (*P230p*) gene sequence.

In silico analysis of SPELD protein sequence across rodent and human *Plasmodium* species

The sequences of SPELD proteins from *P. berghei* (PbSPELD, PBANKA_0910900), *P. chabaudi* (PcSPELD, PCHAS_0712200), *P. yoelii* (PySPELD, PY17X_0912300), *P. cynomolgi* (PcySPELD, PCYB_094370), *P. falciparum* (PfSPELD, PF3D7_1137800), *P. knowlesi* (PkSPELD, PKNH_0936000), and *P. vivax* (PvSPELD, PVX_092505) were obtained from the PlasmoDB. Sequence alignment was performed using the Clustal Omega program (http://www.ebi.ac.uk/Tools/msa/clustalo/). PbSPELD sequence was analysed using the Pfam (http://pfam.xfam.org/), conserved domain database (http://www.ncbi.nlm.nih.gov/Structure/cdd/wrpsb.cgi) and the non-redundant protein sequences database for the presence of conserved domains and non-*Plasmodium* homologs. Transmembrane regions were predicted using multiple programs (TMHMM, TMpred, DAS, TopPred2), and amino acid composition was determined using the ProtParam tool (http://web.expasy.org/protparam/). The PbSPELD sequence was analysed for the presence of motifs using the Motif Scan tool (http://myhits.isb-sib.ch/cgi-bin/motif_scan).

Generation of *P. berghei* ANKA WT GFP line

To achieve a successful DCO homologous recombination, sequences corresponding to fragment A and B within the *P230p* ORF were cloned on either ends of the GFP-hDHFR cassette using XhoI/ClaI and NotI/AscI sites respectively. The targeting construct was separated from vector backbone by digesting with XhoI/AscI and electroporated into the schizont stages. Genomic DNA was isolated from drug resistant GFP positive parasites and correct site-specific integration at fragment A and B was confirmed by primers designed at sites beyond recombination. A diagnostic PCR was performed to amplify product of 890 bp using primer set FP12/RP12 and product of 1140 bp using primer set FP13/RP13, that confirmed the correct integrations at region A and B within the *p230p* locus. Following limiting dilution, the clonal lines were confirmed for successful disruption of the gene by diagnostic PCR using primer set FP11/RP11 that amplified a PCR product of 869 bp only from WT genomic DNA.

Generation of pBC-mCherry-hDHFR reporter plasmid

mCherry open reading frame was amplified using forward primer FP-5'ATACTCGAGATGGTGAGCAAGGGCGAG3' (XhoI) and reverse primer, RP-5'ACCACTAGTTTACTTGTACAGCTCGTCC3' (SpeI) using vector PL0017 as a template.

The PCR product was sequenced and confirmed. The mCherry ORF with restriction sites XhoI and SpeI was cloned in pTZ57R/T vector. A 3' regulatory sequence of HSP70 was PCR amplified using forward primer, FP-5' TATACTAGTTTATTGTTCTGTACTTCTTTT3' (SpeI site underlined) and reverse primer, RP-5'ACTCCCGGGAAAATACCAATAATACCGTTT3' (XmaI site underlined) from pBC-GFP-hDHFR vector and following sequence confirmation, this fragment was cloned into the pTZ57R/T in tandem to mCherry ORF using restriction sites SpeI and SmaI/XmaI. The mCherry ORF along with HSP70 3' UTR was released from pTZ57R/T vector and cloned into pBC-GFP-hDHFR vector using XhoI and XmaI replacing GFP cassette. The vector was named pBC-mCherry-hDHFR.

Labeling and microarray hybridization

The samples for gene expression were labeled using Agilent Quick-Amp labeling Kit (p/n5190-0442). Total RNA were reverse transcribed at 40°C using oligodT primer tagged to a T7 polymerase promoter and converted to double stranded cDNA. Synthesized double stranded cDNA were used as template for cRNA generation. cRNA was generated by *in vitro* transcription and the dye Cy3 CTP (Agilent) was incorporated during this step. The cDNA synthesis and *in vitro* transcription steps were carried out at 40°C. Labeled cRNA was cleaned up using Qiagen RNeasy columns (Qiagen).

Hybridization and Scanning

The labeled cRNA sample were fragmented at 60°C and hybridized on to an Agilent Custom *Plasmodium berghei* Gene Expression Microarray 4x44k designed by Genotypic Technology Private Limited (AMADID No: 067226). Fragmentation of labeled cRNA and hybridization were done using the gene expression hybridization kit (Agilent Technologies, In situ Hybridization kit). Hybridization was carried out in Agilent's surehyb chambers at 65° C for 16 hrs. The hybridized slides were washed using Agilent gene expression wash buffers (Agilent Technologies) and scanned using the Agilent Microarray Scanner (Agilent Technologies) at 5 micron resolution. Data extraction from Images was done using Agilent Feature Extraction software.

Microarray data analysis

Feature extracted raw data was analyzed using Agilent GeneSpring GX software. Normalization of the data was done in GeneSpring GX using the 75th percentile shift method. Percentile shift normalization is a global normalization, where the locations of all the spot intensities in an array are adjusted. This normalization takes each column in an experiment independently, and computes the nth percentile of the expression values for this array, across all spots (where n has a range from 0-100 and n=75 is the median). It subtracts this value from the expression value of each entity and fold change values were obtained by comparing test samples with respect to specific control samples. Significant genes upregulated fold> 0.8 (logbase2) and downregulated <-0.8 (logbase2) in the test samples with respect to control sample were identified. Statistical student T-test p-value among the replicates was calculated based on volcano Plot Algorithm. Differentially regulated genes were clustered using hierarchical clustering based on Pearson coefficient correlation algorithm to identify significant gene expression patterns. Genes were classified based on functional category and pathways using Biological Analysis tool DAVID (http://david.abcc.ncifcrf.gov/).
